# Treatment data using a topical povidone-iodine antiseptic in patients with superficial skin abscesses

**DOI:** 10.1016/j.dib.2019.103715

**Published:** 2019-03-07

**Authors:** Gillian Schmitz, Lauren Rosenblatt, Nicholas Salerno, Julie Odette, Ronnie Ren, Tatiana Emanuel, Joel Michalek, Qianqian Liu, Liem Du, Koursoh Jahangir, Adriana Segura Olson

**Affiliations:** a Uniformed Services University of the Health Sciences, Department of Military and Emergency Medicine, 4301 Jones Bridge Rd, Bethesda, MD 20814, USA; b Department of Emergency Medicine, University of Texas Health San Antonio, 7703 Floyd Curl Mail Code 7736, San Antonio, TX 78229, USA; c University Health System, 4501 Medical Drive, San Antonio, TX 78229, USA; d Department of Medicine, Section of Emergency Medicine, University of Cgicago, 5841 South maryland Ave, MC 5068, Chicago, IL, 60637, USA

## Abstract

The standard treatment of cutaneous abscesses in the emergency department is incision and drainage (I&D). The purpose of this investigation is to determine the feasibility of using a povodine-iodine topical antiseptic solution (PVP-I) as a clinical adjunct in the treatment of superficial skin abscesses after I&D, and the data is related to “Pilot Study to Evaluate the Adjunct Use of a Povidone-Iodine Topical Antiseptic in Patients with Soft Tissue Abscesses” [Olson et al., 2019].

The data aims to determine if the daily application of PVP-I in the wound cavity and as an antiseptic hand wash would confer any benefit over I&D alone. The primary outcome was clinical cure 7–10 days after I&D. The secondary outcomes were rate of new abscess development and spread of infection in household contacts (HC) within 30 days.

**Table:** Specifications table

Subject area	*Microbiology*
More specific subject area	*Abscess, wound care, MRSA, incision and drainage, clinical cure rates*
Type of data	*Table, figure*
How data was acquired	*Patients were screened in the emergency department if they had a skin abscess. Patients who met criteria were enrolled. Clinical data was collected including demographic data and location and size of abscess. Would cultures were collected by the treating physician. Healing rates, adverse events, and wound measurements were collected by an investigator at 7–10 days following the initial incision and drainage. Patients were called at 30 days to determine if they had a recurrence of their skin abscess.*
Data format	*Analyzed*
Experimental factors	*Topical povodine-iodine solution antiseptic solution*
Experimental features	*Randomized controlled trial to determine clinical effectiveness of a topical antiseptic agent in addition to incision and drainage in the treatment of cutaneous abscesses*
Data source location	*San Antonio, TX*
Data accessibility	*Data is in this article*
Related research article	**Olson A, Rosenblatt L, Salerno N. et al., Pilot Study to Evaluate the Adjunct Use of a Povidone-Iodine Topical Antiseptic in Patients with Soft Tissue Abscesses. Journal of Emergency Medicine,** (in press).

**Table:** 

**Value of the data** • Povidone-iodine (PVP-I) solutions have bactericidal activity and have been used as an antiseptic hand wash and irrigation in surgical wounds to decrease infections. The data in this article is important to future researchers and the scientific community to determine if a clinical benefit or harm exists. • This provides pilot data to determine baseline healing rates and treatment failure rates to power a large study. The results of this data can be used as benchmarking data for future collaborative research and to estimate sample size analyses for larger studies. • This data set contains information on common adverse events. The results of this data are useful to the scientific community in order to anticipate potential risks that can occur and should be included in consent documents and protocols for future studies.

## 1 Data

105 patients were evaluated for enrollment. 4 patients were excluded: two patients declined enrollment, one patient was discovered to be an intravenous drug user, and one patient required admission to the hospital for surgical debridement and irrigation in the operating room for a complicated abscess. Of the 101 patients enrolled, 52 were randomized to PVP-I and 49 were randomized to standard treatment. Of the patients randomized to PVP-I, 51 (98%) completed 7–10 follow up and 39 patients (75%) completed 30 day follow up. Of the patients randomized to standard treatment, 45 (92%) completed 7–10 follow up and 39 patients (80%) completed 30 day follow up (See Table 1, Table 2, Table 3, Table 4).

**Table 1:** Baseline demographics of participants (Adapted from Olson et al [1]).

Characteristics	PVP-I (%) N = 52	Standard Care (%) N = 49
Male	33 63.4)	30 (61.2)
Age (mean ± SD)	41.33 ± 14.82	41.76 ± 13.57
Race or Ethnicity		
Hispanic	38 (73.1)	35 (71.4)
White	33 (63.5)	35 (71.4)
Black or African American	9 (17.3)	3 (6.1)
**Culture results**		
MRSA	17 (38.6)	17 (36.2)
MSSA	6 (13.6)	10 (21.3)
**Abscess location**		
Trunk	15 (28.8)	12 (24.5)
Extremity	17 (32.7)	14 (28.6)
Groin/buttock	14 (26.9)	10 (20.4)
Axilla	5 (9.6)	6 (12.2)
Head and neck	6 (11.5)	5 (10.2)
**Cellulitis, cm**		
Median (IQR)	6 (1.25–12.5)	5 (0–9)
Range	0–25	0–28
**Abscess size, cm**^**2**^		
Median (IQR)	18 (8–27)	12 (5–33)
Range	1–180	2–126.5

**Table 2:** Primary outcome: clinical cure 7–10 after drainage (Adapted from Olson et al. [1]).

	Standard Care (%) n = 46	PVP-I (%) n = 51	Difference (95% CI)	*P* Value
Total Clinical Cure at 7–10 Days	42 (91.3%)	45 (88.2%)	3.1 (−10.7, 16.8)	0.53
Clinical Cure at 7–10 Days in Patients on Antibiotics	(n = 34)	(n = 35)		
	31 (91.2%)	31 (88.6%)	2.6 (−14.2, 20.0)	0.53
Clinical Cure at 7–10 Days in Patients Not on Antibiotics	(n = 12)	(n = 16)		
	11 (91.7%)	14 (87.5%)	4.2 (−29.0, 31.0)	0.78

**Table 3:** Secondary outcomes: development of new abscesses in patients and spread in household contacts within 30 days (Adapted from Olson et al. [1]).

	Standard Care (%) (n = 41)	PVP-I (%) (n = 39)	Difference (95% CI)	*P* Value
Development of New Abscesses	8 (19.5%)	8 (20.5%)	−1.0 (−19.4,18.0)	0.96
Spread to Household Contacts	4 (9.7%)	2 (5.1%)	4.6 (−9.1, 19.1)	0.53

**Table 4:** Reported side effects and adverse events (Adapted from Olson et al. [1]).

Characteristics	Standard Care n = 49	PVP-I n = 52	*P*-Value
**Total Reported Adverse Events**	13 (26.5%)	31 (59.6%)	<0.001
Burning/pain	1 (2%)	18 (34.6%)	<0.001
Pruritis	2 (4.1%)	7 (13.5%)	0.11
Tape irritation	7 (14.3%)	7 (13.5%)	0.95
Skin irritation around wound	0	4 (7.7%)	0.05
Skin Discoloration	0	1 (1.9%)	0.51
Chills	0	1 (1.9%)	0.51
Cough	0	1 (1.9%)	0.51
Diarrhea	0	1 (1.9%)	0.51
Decreased Appetite	1 (2%)	0	0.36
Dizziness	1 (2%)	0	0.36
Rash	2 (4.1%)	0	0.16

## 2 Experimental design, materials, and methods

A pilot study was carried out, including a randomized controlled trial of adult patients with an uncomplicated skin abscess. All patients had their abscesses incised and drained. Patients were randomized to either PVP-I or no additional treatment (standard care). Patients randomized to PVP-I were instructed on daily application of the agent to hands, wound, and surrounding skin with wound care and dressing changes. Patients randomized to standard treatment were instructed on hand washing with soap and water and wound care with dressing changes. Subjects were asked to return at 48–72 hours and 7–10 days for a wound recheck. Patients were also followed up by phone at 30 days. The primary outcome was clinical cure 7–10 days after I&D. The secondary outcomes were rate of new abscess or skin lesions within 30 days and spread in household contacts (HC) within 30 days.

Inclusion criteria were patients 18 years and older presenting to the ED or urgent care center with an uncomplicated skin abscess requiring I&D. Exclusion criteria were patients who were unable to provide informed consent, were homeless or incarcerated, had active intravenous drug use or iodine allergy, had an abscess on the breast or face, or required inpatient admission to the hospital or surgical drainage. Patients were randomized in blocks of 10 to PVP-I or standard care. Patients were allocated to each arm after opening sequentially numbered, sealed envelopes.

### 2.1 Methods of measurement

Standard demographic data was collected on all enrolled patients, including contact information, age, gender, ethnicity, location of abscess (es), length and width of palpable fluctuance and induration, length and width of cellulitis, presence or absence of fever, and previous medical history. Measurements of the abscess and surrounding cellulitis were measured using a ruler provided in each enrollment packet.

### 2.2 Interventions

All abscesses were incised, drained, and irrigated according to standard practice. Abscess cavity wound cultures were obtained and sent for bacterial cultures and antibiotic susceptibility testing.

Patients randomized to PVP-I had the abscess cavity and surrounding skin gently painted with the PVP-I solution with a cotton tip applicator. The contents of one foil packet of PVP-I were applied to the walls and floor of the abscess cavity by a research associate after drainage was performed. The contents of a second foil packet were applied to the surrounding skin within 5 cm around the incision. The abscess cavity was then gently packed with ¼ inch plain gauze strips and the wound was covered with 4 × 4 gauze and secured with tape.

Patients randomized to standard care had the abscess cavity packed with ¼ inch plain gauze strips after incision and drainage and the wound was covered with 4 × 4 gauze and secured with tape.

Patients assigned to the treatment group received instructions on how to use and apply PVP-I as part of their daily wound care routine and patients assigned to the standard of care group were advised about dressing changes and hand washing with soap and water.

Patients in both groups were instructed to leave the wound packing in place and change the outer dressing as needed or at least once a day until they returned at 48–72 hours for their first wound recheck. Providers were discouraged from prescribing routine antibiotics unless the clinician felt it was clinically indicated.

### 2.3 Wound rechecks and follow up visits

Subjects returned for an initial wound recheck within 48–72 hours of the I&D for data collection. Patients randomized to the PVP-I arm had the internal packing removed, PVP-I reapplied to the walls of the abscess cavity and surrounding skin, and instructed on wound care and hand hygiene. Patients were taught to first apply one foil packet of PVP-I themselves to their hands and fingers as a protective barrier. They were then instructed to apply the contents of a second foil packet to the walls and floor of the abscess cavity using a cotton-tipped applicator. Patients were then instructed to apply a third foil packet to the skin surrounding the abscess within a 5cm diameter of the wound using a separate cotton-tipped applicator. Patients were instructed to gently wash their hands and cover the wound with a 4 × 4 gauze dressing. An investigator watched each patient perform this routine once in the emergency department to ensure the patient understood how to apply PVP-I at home.

Patients randomized to standard care had the internal packing removed and the wound covered with a 4 × 4 gauze dressing.

Patients in both arms were instructed not to pack the wound cavity at home. All patients were instructed to remove the outer dressing at home and soak in water once a day for at least 2–3 minutes. They were instructed to gently massage the skin around the abscess in the bath or shower to allow further drainage of the wound.

Patients randomized to PVP-I were instructed to perform their wound care routine once daily by applying the topical agent with the three foil packets, as described above, until they were seen for their second wound recheck (7–10 days) or until wound cavity had closed. Patients also were instructed to keep a storage bag of used PVP-I packets to assess compliance at the follow up visits.

All patients were instructed to cover the abscess cavity with a 4 × 4 dry dressing after wound cleaning and change the dressing as often as needed if it became saturated. Subjects were asked to return at 7–10 days after enrollment for a second wound check. Research personnel contacted patients by telephone for data collection at 30 days after enrollment.

### 2.4 Data collection

Investigators recorded clinical data that included the presence or absence of clinical cure, need for additional intervention, compliance with the PVP-I, and rate of adverse events at both return visits. Compliance with wound care was assessed by measuring the number of opened foil packets that patients returned at their wound rechecks and by patient report. Patients were considered compliant with PVP-I if they used and returned all of the packets given to them. Data were collected regarding the presence of new lesions and spread in household contacts during each follow-up visit and at the 30 day follow up phone call.

### 2.5 Outcome measures

The primary outcome was clinical cure 7–10 days after I&D. Clinical cure was defined as improvement in the initial wound with respect to a decrease in measured size, erythema, and purulent discharge without need for further clinical intervention. Wound management at the follow-up visits was left up to the discretion of the treating provider, but additional interventions for patients not clinically improving or worsening were considered a lack of clinical cure. The secondary outcomes were rate of development of skin abscesses in household contacts and rate of recurrent skin lesions in patients treated with incision and drainage. A new lesion was defined as a new abscess, pustule, carbuncle, or furuncle at least 5cm away from the initial wound. Lesions within 5cm of the initial wound were considered failures of the initial abscess treatment and were considered a lack of clinical cure.

### 2.6 Statistical analysis

Categorical outcomes were summarized with the sample size, mean, and standard deviation and frequencies and percentages of continuously distributed outcomes were reported. The significance of variation in the difference of proportions with treatment (PVP-I, Control) was assessed with the exact distribution of the binomial and variation in the mean with treatment was assessed with T-tests. All statistical testing was two-sided with a significance level of 5%. SAS Version 9.4 and StatXact Version 11 for Windows were used throughout.

## References

[1] Olson A., Rosenblatt L., Salerno N. (2019 Feb 27). Pilot study to evaluate the adjunct use of a Povidone-iodine topical antiseptic in patients with Soft Tissue abscesses. J. Emerg. Med..

