# Supplementary material for: Treatment data using a topical povidone-iodine antiseptic in patients with superficial skin abscesses
Source: Data Brief. 2019 Mar 7;23:103715. doi: 10.1016/j.dib.2019.103715 (PMC6660432; doi:10.1016/j.dib.2019.103715)
Supplement: Multimedia component 1 [file mmc1.docx]

Funding for this research study was provided by the Microdermis Corporation. Microdermis was involved in the study design, but had no involvement in the collection, analysis, or interpretation of the data or the writing of this manuscript. I had full access to all of the data in this study and I take responsibility for the integrity of the data and the accuracy of the data analysis.

The study that this data in brief references was recently accepted for publication in the Journal of Emergency Medicine. The data presented here summarizes the relevant information but has not been previously published in its current form and is not currently under consideration at another journal. The tables and manuscript have been adapted as requested and will cite the original article once the full citation is available.

Corresponding Author Contact Information:

Gillian R. Schmitz, MD, FACEP

Department of Military and Emergency Medicine

Uniformed Services University of the Health Science

4301 Jones Bridge Rd, Bethesda, MD 20814

[GillianMD@gmail.com](mailto:GillianMD@gmail.com)

(210) 542-7783
